# Contribution of ATPase copper transporters in animal but not plant virulence of the crossover pathogen *Aspergillus flavus*

**DOI:** 10.1080/21505594.2018.1496774

**Published:** 2018-08-23

**Authors:** Kunlong Yang, Yana Shadkchan, Joanna Tannous, Julio A. Landero Figueroa, Philipp Wiemann, Nir Osherov, Shihua Wang, Nancy P. Keller

**Affiliations:** a Fujian Key Laboratory of Pathogenic Fungi and Mycotoxins, Key Laboratory of Biopesticide and Chemical Biology of Education Ministry, and School of Life Sciences, Fujian Agriculture and Forestry University, Fuzhou, China; b Department of Medical Microbiology and Immunology, University of Wisconsin, Madison, WI, USA; c Aspergillus and Antifungal Research Laboratory, Department of Clinical Microbiology and Immunology, Sackler School of Medicine, Tel Aviv University, Tel Aviv, Israel; d Agilent Metallomics Center, College of Arts & Science, Chemistry Department, University of Cincinnati, Cincinnati, OH, USA

**Keywords:** P_1_-type ATPase, ROI stress, copper homeostasis, AceA, CrpA, CrpB

## Abstract

The ubiquitous fungus *Aspergillus flavus* is notorious for contaminating many important crops and food-stuffs with the carcinogenic mycotoxin, aflatoxin. This fungus is also the second most frequent *Aspergillus* pathogen after *A. fumigatus* infecting immunosuppressed patients. In many human fungal pathogens including *A. fumigatus*, the ability to defend from toxic levels of copper (Cu) is essential in pathogenesis. In *A. fumigatus*, the Cu-fist DNA binding protein, AceA, and the Cu ATPase transporter, CrpA, play critical roles in Cu defense. Here, we show that *A. flavus* tolerates higher concentrations of Cu than *A. fumigatus* and other *Aspergillus* spp. associated with the presence of two homologs of *A. fumigatus* CrpA termed CrpA and CrpB. Both *crpA* and *crpB* are transcriptionally induced by increasing Cu concentrations via AceA activity. Deletion of *crpA* or *crpB* alone did not alter high Cu tolerance, suggesting they are redundant. Deletion of both genes resulted in extreme Cu sensitivity that was greater than that following deletion of the regulatory transcription factor *aceA*. The Δ*crpA*Δ*crpB* and Δ*aceA* strains were also sensitive to ROI stress. Compared to wild type, these mutants were impaired in the ability to colonize maize seed treated with Cu fungicide but showed no difference in virulence on non-treated seed. A mouse model of invasive aspergillosis showed Δ*crpA*Δ*crpB* and to a lesser degree Δ*aceA* to be significantly reduced in virulence, following the greater sensitivity of Δ*crpA*Δ*crpB* to Cu than Δ*aceA.*

## Introduction

Copper (Cu) is an essential metal serving as a cofactor for enzymes that function in numerous biological processes in both prokaryotes and eukaryotes [1,2]. The property of Cu to have both reductive (Cu^+^) and oxidative (Cu^2+^) states is important in several biological functions [1]. For example, the reducing ability of Cu^+^ is critical for the activities of Cu-dependent enzymes like Cu/Zn superoxide dismutase (SOD), cytochrome oxidase and laccase [3]. On the other hand, high concentrations of Cu are toxic due to Fenton chemistry, by which Cu can generate harmful reactive oxygen species such as hydroxyl radicals [4,5]. Cu also has a high affinity to displace other metals from their cognate proteins leading to protein inactivation [2,6]. Thus, Cu homeostasis is tightly controlled by cells.

Since Cu leads to toxic oxidative stress, which could cause DNA damage and protein dysfunction, Cu as a sole source or in mixtures such as the Bordeaux mixture has been used as an antimicrobial agent to kill plant pathogens, employed to sterilize wounds or to treat surfaces both external or internal (e.g. implants) susceptible to microbial contamination [7–10]. The anti-microbial properties of Cu are also used by mammalian host cells as defense mechanisms. For example, Cu plays an important role in innate immune defense to microbial infection in mammals [8,11,12]. Previous studies demonstrated that, upon activation by a pathogen, macrophages elevate the expression levels of high-affinity Cu^+^ importer Ctr1 and P-type Cu ATPase, ATP7A, leading to increased levels of Cu in the phagosome of IFN-γ stimulated macrophages [13,14]. These high Cu levels, together with Fe could react chemically with ROIs and reactive nitrogen intermediates (RNIs), potentially magnifying the Cu mediated toxicity.

On the other hand, Cu homeostasis has been shown to support the virulence of many fungal pathogens, which share many of the elements found in mammalian Cu regulatory systems including Cu-binding transcriptional factors (TFs), Cu-importers/exporters and Cu-metallothioneins (MTs)[2,15]. Fungal Cu-binding TFs tightly control expression levels of genes involved in Cu uptake and export under Cu-deficient conditions or Cu-excess respectively. In *Saccharomyces cerevisiae*, Cu importers Ctr1 and Ctr3, located in the plasma membrane, as well as the Cu-MTs Fre1 and Fre7 are activated by the Cu-binding TF Mac1 under Cu-insufficient conditions [8,16]. Under excess Cu, the *S. cerevisiae* Cu-binding TF Ace1 induces the MT-encoding genes *CUP1* and *CRS5*, and the Cu/Zn SOD-encoding gene *SOD1* [8,17]. In the human pathogen *Cryptococcus neoformans*, MTs play a major role of in Cu detoxification [7]. Two MTs, Cmt1 and Cmt2, were shown to be important for fungal virulence in *C. neoformans*, both of which are regulated by the Cu-binding TF Cuf1 [2,18]. In *Candida albicans*, a membrane-localized P-type Cu ATPase, Crp1, is responsible for Cu resistance along with the MT Cup1, both of which are transcriptionally activated by Ace1 under high Cu conditions [19–21]. Recent studies also illustrated the regulation of Cu homeostasis in the filamentous fungi *Aspergillus fumigatus* and *A. nidulans*, both of which possess the same Cu export machinery [15,22,23]. In *A. fumigatus* and *A. nidulans*, the Cu-fist binding TF AceA senses high levels of Cu, and induces the Cu P-type ATPase CrpA as a detoxification mechanism [15,22]. Both AceA and CrpA are virulence factors for the human pathogenic *A. fumigatus*. Currently there are no studies of the roles of P-type ATPases in plant pathogenic fungi although a CCC-2 type ATPase, BcCCC2, important in melanization in *Botrytis cinerea* is necessary for fungal development and virulence of that pathogen [24]. The BcCCC2 homolog in *A. fumigatus*, CtpA, is also required for spore melanin synthesis in that fungus [25].

The ubiquitous fungus *Aspergillus flavus* is the second most common *Aspergillus* spp. to infect immunosuppressed patients after *A. fumigatus* [26] as well as being a frequent cause of trauma-associated keratitis in agricultural workers [27]. *Aspergillus flavus* is also notorious for its ability to colonize lipid-rich seed resulting in contamination of food and feed crops with the carcinogenic mycotoxin, aflatoxin [28] This dual ability to cause disease in both animals and plants, leading to enormous agricultural economic losses and health problems, exacerbates the importance of *A. flavus* as an opportunistic pathogen.

As we have recently found that deletion of the Cu export machinery severely impairs *A. fumigatus* virulence [15], we queried if the same proteins were also important for virulence of *A. flavus*, both in its role as a plant and mammalian pathogen. Unexpectedly we found that *A. flavus* possesses two copies of the Cu exporter P1-type ATPase, CrpA and CrpB which results in greater tolerance of Cu in this species than other Aspergilli. We demonstrate that both *crpA* and *crpB* are transcriptionally induced by the Cu-fist TF AceA. Only the double deletion of *crpA* and *crpB* resulted in a strain sensitive to low concentrations of Cu, indicating a redundant function of these two proteins. Neither the Δ*crpA*Δ*crpB* or Δ*aceA* mutants showed any defect in colonizing host maize seed but, as expected, both strains failed to colonize maize corn treated with Cu fungicide. On the other hand, the Δ*crpA*Δ*crpB* mutant was significantly reduced in virulence in a murine model of invasive aspergillosis with loss of AceA showing a notable but lesser influence on fungal invasion. This difference in murine virulence between these two mutants reflects the relative sensitivity of these mutants to Cu concentrations.

## Results

### Aspergillus flavus and A. parasiticus tolerate higher concentrations of Cu

**Figure 1.**
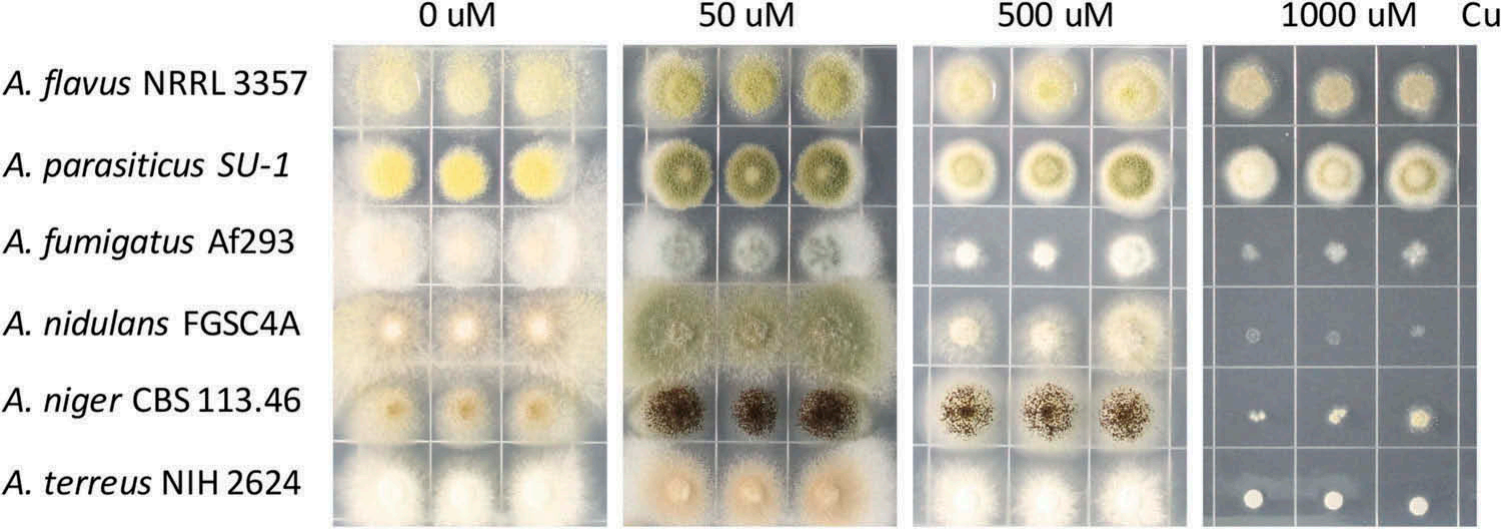
Growth phenotype of different *Aspergillus* strains on different Cu concentrations. 2000 spores of indicated *Aspergillus* strains grown on solidified GMM under indicated Cu concentrations for 72 h at 37°C.

Most *Aspergillus* species are considered as soil-borne fungi living in environments with fluctuating levels of heavy metals including Cu. This is particularly true for the agricultural plant pathogens *A. flavus* and *A. parasiticus* which may be found in fields treated with Cu fungicides [29]. To test whether *Aspergillus* species exhibit similar growth patterns to Cu extremes, six *Aspergillus* species (*A. flavus* NRRL3357, *A. parasiticus* SU-1, *A. fumigatus* Af293, *A. nidulans* FGSC4A, *A. niger* CBS 113.46 and *A. terreus* NIH2624) were selected and grown on GMM media supplemented with different concentrations of Cu. In all species, Cu is a cofactor for laccases required for conidia pigmentation and thus, as expected, all species could not produce normal pigment without Cu (Figure 1). The sensitivity of *A. fumigatus* became apparent at 500 μM Cu on GMM medium after 3 days (Figure 1). *Aspergillus nidulans, A. niger* and *A. terreus* exhibited total growth inhibition at 1000 μM Cu, while *A. flavus* and *A. parasiticus* remained quite tolerant to this level of Cu (Figure 1).

We were curious to see if this enhanced resistance to copper was a general feature of *A. flavus* and *A. parasiticus*. We thus grew four *A. oryzae* strains (a clade of *A. flavus*), five more *A. flavus* strains, four more *A. parasiticus* strains, five more *A. fumigatus* strains and then a few other *Aspergillus* species. Growth phenotypes of all *A. oryzae, flavus* and *parasiticus* strains at 1 mM copper was only almost unaltered compared to lower concentrations, while none of the *A. fumigatus* strains grew at this copper concentration (Figure S1). Of the five other species assessed, two, *A. carbonarius* and *A. brasiliensis* also tolerated 1 mM Cu. Intriguingly, when the concentration of Cu was increased to 2 mM, both *A. carbonarius* and *A. brasiliensis* could still grow, but slightly less than at lower concentrations, while the tested *A. oryzae, flavus* and *parasiticus* strains showed almost no growth impairment (Figure S2).

### Aspergillus flavus and A. parasiticus possess a redundant copy of the P type Cu-exporting atpase

**Figure 2.**
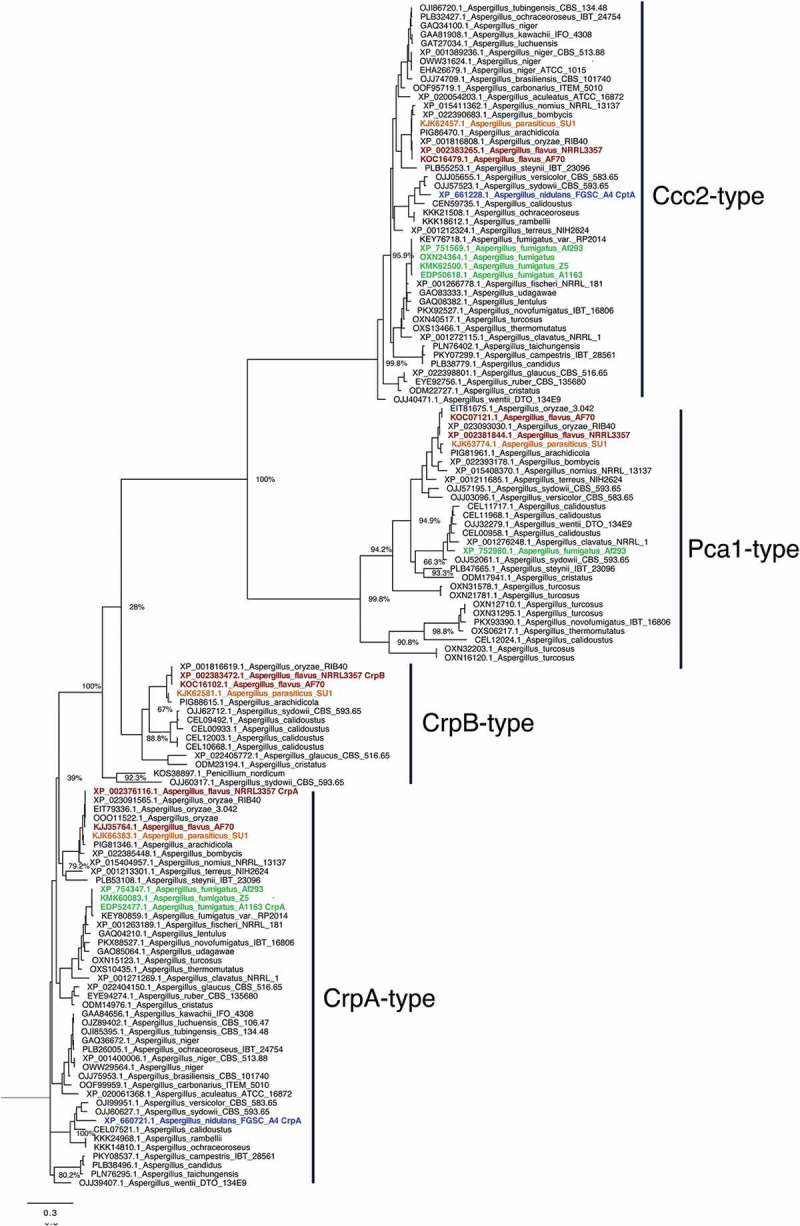
Phylogenetic analysis of heavy metal ATPases (HMA). The phylogenetic tree based on the available HMA sequences from *Aspergillus* sequences available through NCBI was constructed by MAFFT and Fasttree using the Neighbour-joining method as described in Material and Methods. Bootstrap analysis was performed with 1000 replicates. *Aspergillus flavus* proteins are shown in red, *A. fumigatus* in green, *A. parasiticus* in orange, and *A. nidulans* in green.

The Cu exporter P1-type ATPase is the primary means for Cu detoxification in *A. fumigatus* [15]. To identify the Cu P1-type ATPase in *Aspergillus* species, the available protein sequences of the heavy-metal ATPases (HMA) from *Aspergillus* species available from the NCBI database were retrieved (http://www.aspgd.org). The *A. fumigatus* CrpA (AfuA_3g12740 [15]) sequence was used to perform a BLASTP [30] analysis. All sequences with an e-value below 1^−20^ were aligned using MAFFT [31]. The conserved E1-E2 ATPase domains were extracted, re-aligned and a phylogenetic tree was created using fasttree [32]. The analysis shows the three distinct groups including Ccc2-type, CrpA-type and Pca1-type ATPases (Figure 2). Surprisingly, we found that *A. flavus* and *A. parasiticus* have two predicted protein sequences (XP_002376116.1/AFL2T_03712/AFLA_020960 and XP_002383472.1/AFL2T_10544/AFLA_053470) in the CrpA clade of Cu-exporting ATPases. As AFL2T_03712 from *A. flavus* fell into a subgroup with CrpA from *A. fumigatus*, we named it CrpA. Consequently, AFL2T_10544 was termed CrpB. Notably, other *Aspergillus* species (*A. arachidicola, A. glaucus*, and *A. cristatus*) also harbor two predicted Crp-type proteins. Interestingly, *A. sydowii* and *A. calidoustus* seem to harbour even more predicted proteins that fall in this group of Cu-exporting ATPases (Figure 2). Some of the analyzed *Aspergillus* species (*A. sydowii, A. turcosus*, and *A. calidoustus*) also show multiple proteins in the Pca1-type group. The Pca1-type group derived its named from the *Saccharomyces cerevisiae* cadmium transporting ATPase Pca1p [33]. This is in contrast to the Ccc2-type group (named after the *S. cerevisiae* Cu transporting ATPase of the trans-Golgi network Ccc2p [34], where only one predicted protein from each analyzed *Aspergillus* species was found (Figure 2).

### CrpA and CrpB play redundant roles in Cu detoxification in A. flavus

**Figure 3.**
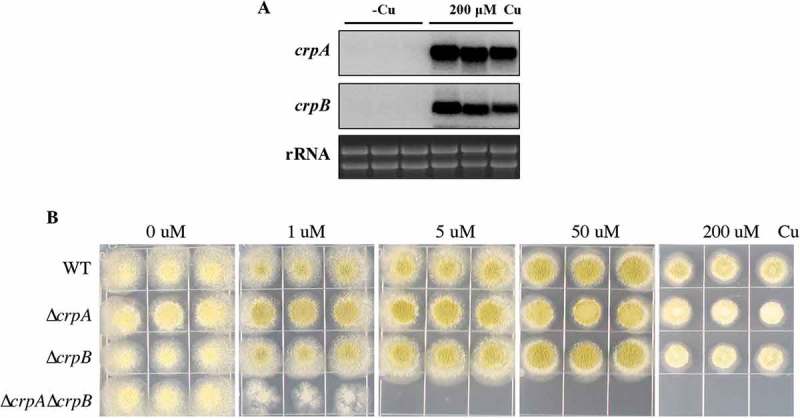
*crpA* and *crpB* were induced by Cu and were redundant in Cu detoxification. (A) Northern blot analysis of *crpA* and *crpB* in *A. flavus* wild-type strain. The wild-type strain was grown in liquid GMM without Cu for 24 h at 37°C and then mycelium mass was divided in half where one-half was grown in medium with no Cu and one-half was grown in 200 μM Cu for 1 h before harvesting. rRNA visualization is loaded as control. (B) Growth assay of *A. flavus crp* mutants on solidified GMM for 72 h at 37°C under indicated Cu concentrations.

Previous studies had demonstrated that the expression level of the Cu exporter CrpA is induced in high levels of Cu in *Aspergillus* spp [15,22]. To determine if both *crpA* and *crpB* are Cu-inducible in *A. flavus*, their expression levels were assessed after *A. flavus* wild-type strain NRRL3357 was treated with 200 μM Cu. As shown in Figure 3A, *crpA* and *crpB* were not transcribed in the absence of Cu, but were strongly expressed under excess Cu treatment.

To test whether CrpA or CrpB affected *A. flavus* Cu tolerance in growth studies, we generated single-deletion mutants of each gene. Diagnostic PCR and Southern analysis were performed to verify correct gene replacement (Figure S3A). Both Δ*crpA* and Δ*crpB* mutants showed wildtype arginine heterotrophy, indicating that the *argB* selectable marker was fully functional and unaffected by its chromosomal location (Figure S4). Both mutants exhibited WT sensitivity to high concentrations of Cu (Figure 3(B)). To determine whether Δ*crpA* and Δ*crpB* are redundant in *A. flavus*, we generated the double deletion mutant Δ*crpA*Δ*crpB*, verified by Southern blot analysis (Figure S3A). As shown in Figure 3(B), the double deletion mutant Δ*crpA*Δ*crpB* was highly sensitive to Cu (1 μM), indicating that CrpA and CrpB have an overlapping function in Cu detoxification.

### The Cu fist transcriptional factor acea regulates crpA and crpB expression during Cu homeostasis

**Figure 4.**
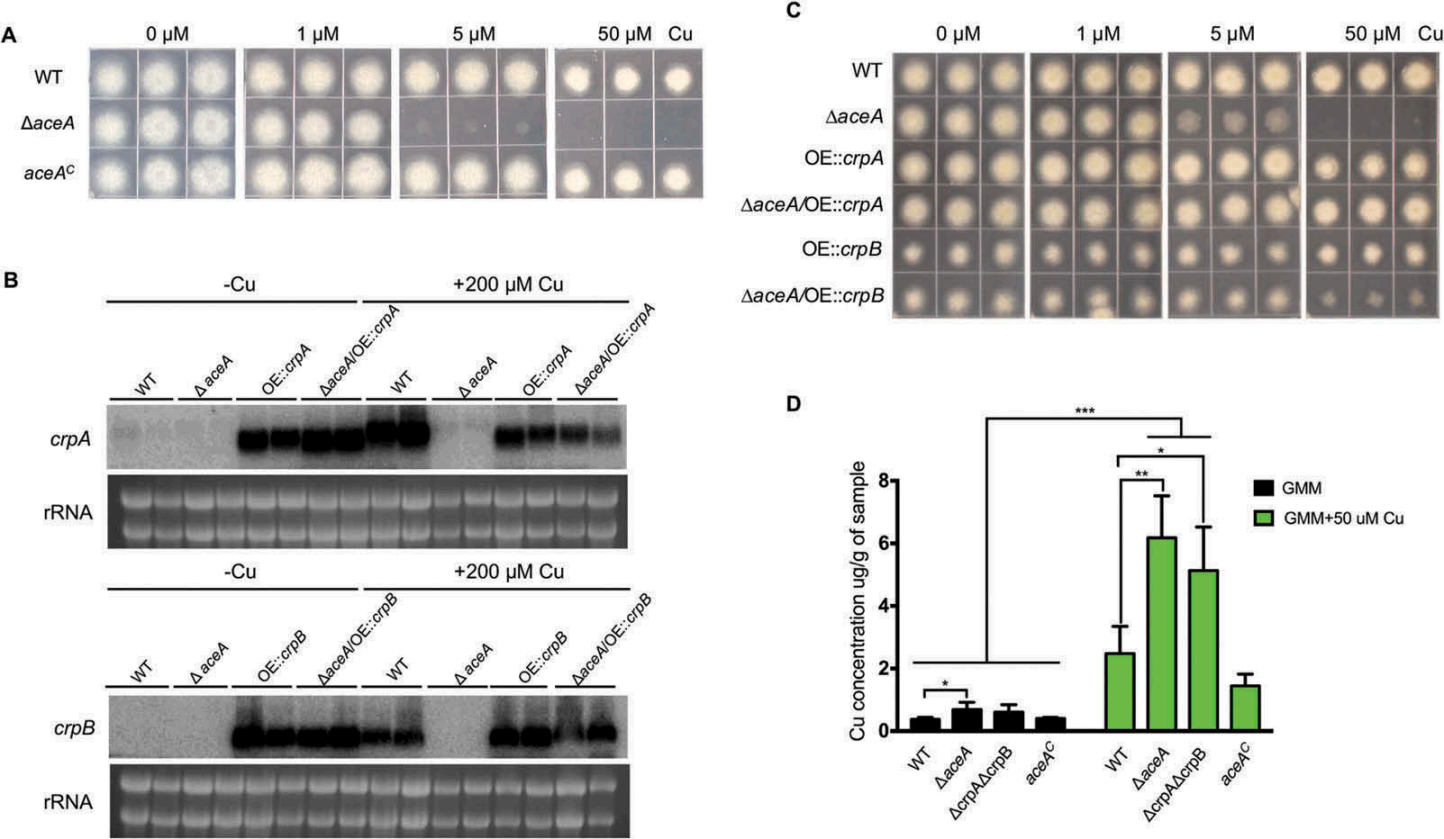
Functional analysis of AceA and its regulation on *crpA* and *crpB*. (A) Growth assay of Δ*aceA* and *aceA^C^* mutants on solidified GMM for 72 h at 37°C under indicated Cu concentrations. (B) Northern blot analysis of *crpA* and *crpB* in *A. flavus* wild-type strain. The wild-type strain grown in liquid GMM without Cu for 24 h at 37°C, then to one-half of the cultures, Cu was added to a final concentration of 200 μM for 1 h before harvesting. rRNA visualization is loaded as control. (C) Phenotypic analysis of the strains on solidified GMM under the indicated Cu concentrations for 48 h at 37°C. (D) Total Cu amount from *A. flavus* mycelia. All strains (5 × 10^6^ conidia/L) were incubated in 50 mL of liquid GMM without Cu in four replicates at 37°C and 200 rpm shaking for 12h. To one-half of the cultures, Cu was added to a final concentration of 50 μM for 12 h before harvesting. Error bars represent standard deviations, asterisk “*, “**” or “***” represent significant differences at p < 0.05, p < 0.01 and p < 0.001, respectively, according to t-test.

The *A. fumigatus* Cu fist DNA binding domain protein AceA regulates responses to excess Cu and activates the Cu exporter CrpA for Cu detoxification [15]. BlastP analyses using the protein sequence of *A. fumigatus* AceA (XP_750669.1) as query allowed the identification of the AceA homolog in *A. flavus*, which is encoded by AFLA_036460 and contains the conserved Cu fist DNA binding domain at its N-terminal (Figure S5). To test its impact on Cu homeostasis in *A. flavus*, the deletion mutant of *aceA* was generated and confirmed by Southern blot analysis (Figure S3A). The Δ*aceA* mutants did not exhibit marker gene effects and one *aceA* mutant (TKY5.3) was used for growth assays as well as for generating complementation strains. The Δ*aceA* mutant exhibited reduced growth on GMM + 5 μM Cu after 3 days (Figure 4A) although it was not as sensitive as the *ΔcrpAΔcrpB* mutant which could not grow at this Cu concentration (Figure 3(B)). Wild-type Cu resistance was restored in the Δ*aceA* complemented strain (Figure 4(A)). These results indicate that AceA plays an important role in regulating Cu homeostasis in *A. flavus*.

To investigate whether the Cu-inducible expression of *crpA* and *crpB* depends on AceA, we tested the expression levels of *crpA* and *crpB* in the *aceA* deficient mutant under excess Cu conditions. Our results showed that expression of *crpB* was not detectable in the Δ*aceA* mutant with *crpA* showing marginal expression in the Δ*aceA* mutant (Figure 4(B)). This demonstrated that AceA is necessary for the activation of *crpA* and *crpB* in Cu detoxification. We next constitutively expressed both *crpA* and *crpB* in the Δ*aceA* mutant and wild-type strain, which were verified first by Southern blot analysis (Figure S3C) and then gene expression confirmed by northern analysis (Figure 4(B)). The growth analysis on GMM medium with high Cu showed that constitutive expression of *crpA* or *crpB* in the Δ*aceA* background largely restored Cu tolerance (Figure 4(C)). Notably, overexpression of *crpB* resulted in a general reduction of fungal growth independent of Cu concentration. Taken together, these data confirm that the *A. flavus* Cu fist TF AceA is responsible for activating genes related to Cu detoxification under Cu excess.

Next, we determined and compared total Cu levels in the mycelium of all four strains. At the Cu depleted condition, the Δ*aceA* mutant showed a significant increase in Cu concentration compared to wild-type strain (Figure 4(D)). While treated with 50 µM Cu, both the Δ*aceA* mutant and the double deletion mutant *ΔcrpAΔcrpB* displayed an excess accumulation of the intracellular Cu levels (Figure 4(D)), which indicated that the abnormal accumulation of Cu in these mutants make them more sensitive to Cu.

### Cu detoxification mediated by AceA increases ROI stress

**Figure 5.**
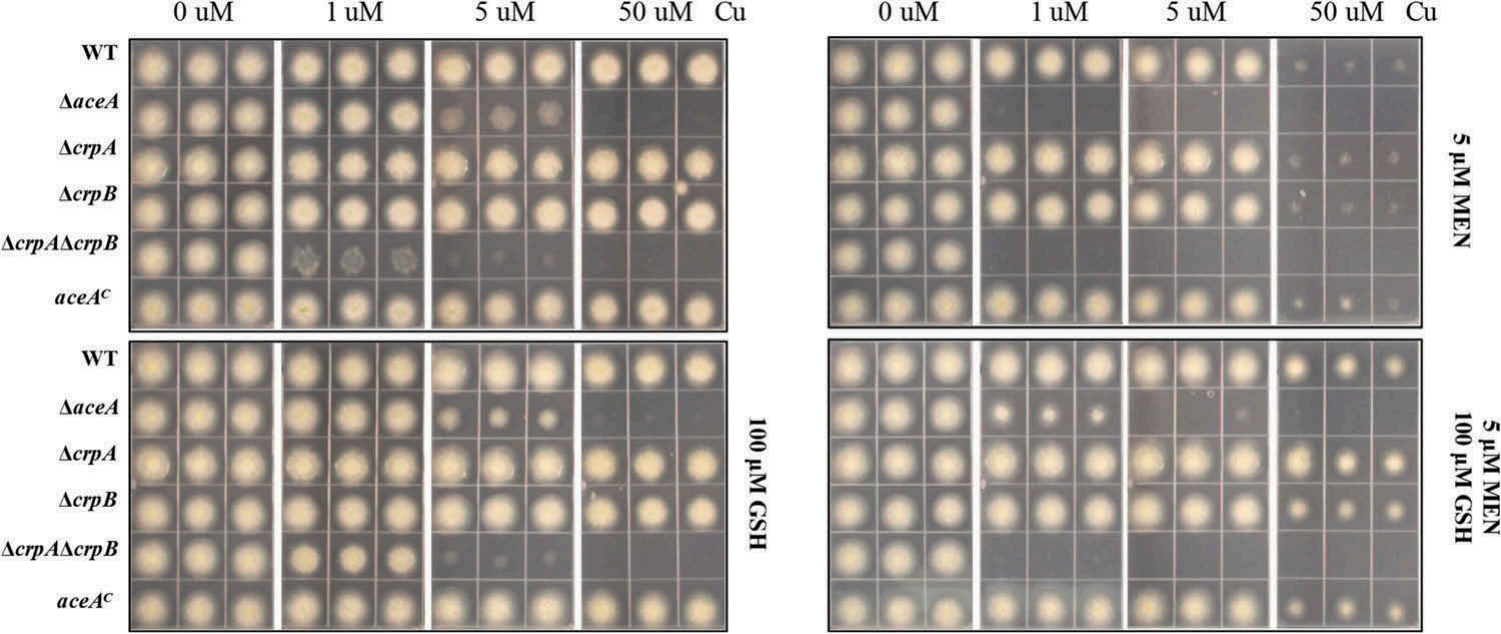
ROS stress increases Cu toxicity in Δ*aceA* and double Δ*crpA*Δ*crpB* mutants. Growth phenotypes of indicated strains on solidified GMM under indicated concentration of Cu plus supplements as indicated for 48 h at 37°C. MEN = menadione, GSH = l-glutathione.

As noted, excess Cu generates reactive oxygen species such as hydroxyl radicals. To test the role of the Cu detoxification machinery in dealing with ROI stress, all of the mutants were incubated on GMM media with increasing Cu and two ROI stressors: the superoxide generator, menadione or hydrogen peroxide. As shown in Figure 5, when grown on 5 μM menadione and increasing Cu, all strains exhibited growth inhibition, with Δ*crpA*Δ*crpB* in general showing the most severe phenotype. This trend was generally similar in hydrogen peroxide treatment although Δ*crpA*Δ*crpB* showed more similar growth to Δ*aceA* (Figure S6A). Addition of the reductant l-glutathione (GSH) in the Cu-containing GMM media, mitigated inhibition in all strains (Figure 5), with suggestion that damage to the fungal Cu detoxification pathway enhances ROI toxicity (more noticeable with hydrogen peroxide treatment and 5 μM copper, Figure S6B).

### Cu fungicide reduces pathogenicity of ∆acea and ∆crpA∆crpB mutants

**Figure 6.**
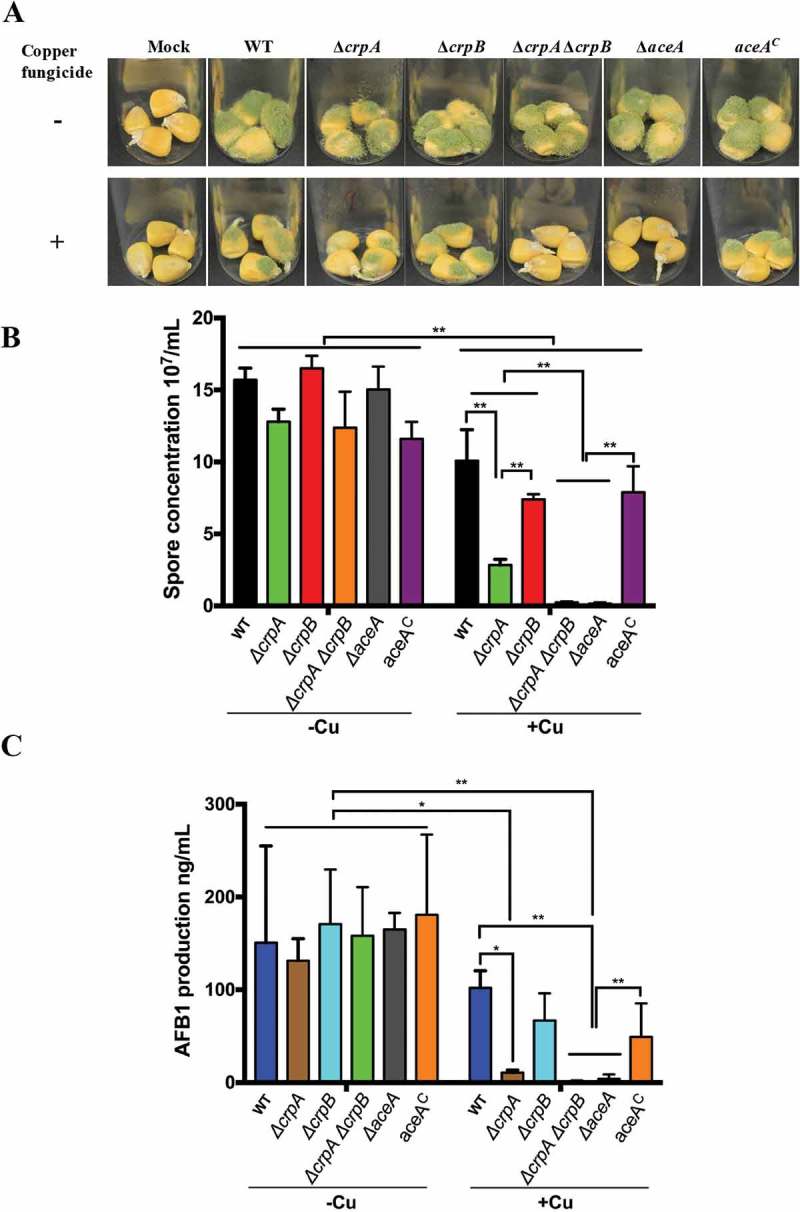
Corn infection with indicated strains. (A) 200 ul of a 10^6^ spore/ml suspension of spores in 0.01% Tween 20 were inoculated on corn and the vials kept in a moist incubator at 29℃ with 12 hours’ light/dark cycling for 5 days. (B) Conidial production assessed from infected maize kernel with and without Cu fungicide treatment. The spores were washed off the seeds with 2.5 mL 100% MeOH and counted. Each sample has four replicates. (C) Aflatoxin extracted from corn and quantified by HPLC. Asterisk “*” or “**” represent significant differences at *p *< 0.01 and *p *< 0.001, respectively, according to t-test.

Aflatoxin contamination of many important seed crops causes enormous agricultural economic losses [35]. Several antimicrobial agents including Cu fungicides have been examined for *A. flavus* growth inhibition as potential post-harvest interventions [36]. Here we examined the *A. flavus* Δ*crpA*Δ*crpB* and Δ*aceA* mutants for their abilities to invade both untreated and Cu fungicide-treated maize kernels. As shown in Figure 6 the mutants grew vigorously on maize seeds not treated with fungicide. However, when the maize kernels were treated with the Cu fungicide (Champion wettable powder), the growth of all the *A. flavus* strains on maize kernels was inhibited (Figure 6(A) lower panel) with statistically less conidia produced than on untreated seed (Figure 6(B,C)). A total growth inhibition was found both in the Δ*aceA* and Δ*crpA*Δ*crpB* mutants on the Cu fungicide treated maize kernels (Figure 6(B,C)).

Of interest, the Δ*crpA* strain, although not showing defects of growth when grown on high Cu-containing media (Figure 3), grew less well and exhibited a significant drop in conidial production compared to the wild-type strain when grown on the treated maize kernels (Figure 6(A,C)). Taken together, these results suggested that the Cu exporters CrpA and CrpB, activated by the Cu-sensing TF AceA, function as Cu defense machinery in hyperaccumulating Cu environments with possibly CrpA playing a slightly more prominent role in Cu export.

### The ΔcrpAΔcrpB mutant is attenuated in virulence in a murine model of invasive aspergillosis

**Figure 7.**
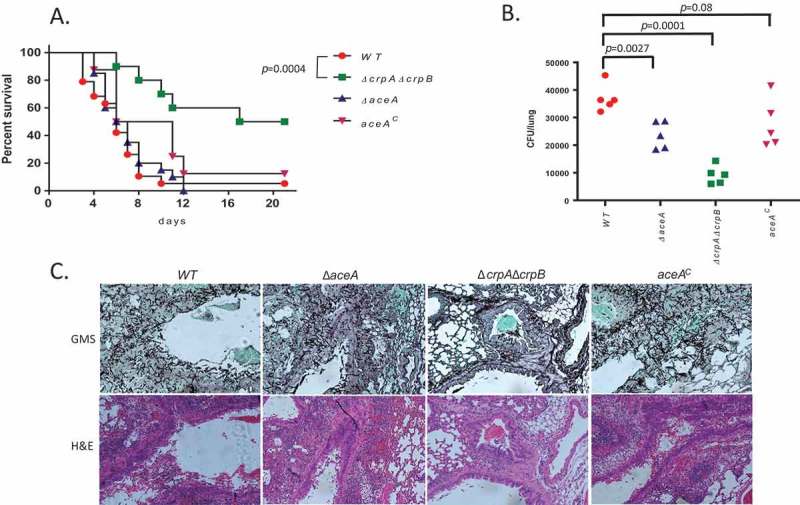
Deletion of *A. flavus crpA* and *crpB* results in attenuated virulence in lung-infected immunocompromised mice. (A) Survival rates of ICR mice immunocompromised with cortisone acetate and infected intranasally with 5 × 10^5^ *A. flavus* WT (n = 19 animals), Δ*crpA*Δ*crpB* (n = 10), Δ*aceA* (n = 19) or *aceA^c^* complemented (n = 8) strains, and survival monitored over 18 days. Virulence was only attenuated in the Δ*crpA*Δ*crpB* strain (*p* = 0.0004) and not in the Δ*aceA* strain (*p* = 0.56). (B) Colony forming unit (CFU) of wild type and copper mutants from infected mice lungs. (C) Histopathology of infected mice lungs stained with Grocott’s methenamine silver stain (GMS; fungal staining) and hematoxylin and eosin (H&E; tissue and nuclear staining). Bar = 200 µm.

We have previously shown that deletion of AceA or CrpA in *A. fumigatus* strongly attenuates virulence in murine models of pulmonary aspergillosis [15]. Here, we tested the virulence of the *A. flavus* WT, Δ*aceA, AceA^c^* and Δ*crpA*Δ*crpB* strains for virulence in a steroid model of invasive pulmonary aspergillosis. Survival curves show that, unlike in *A. fumigatus*, Δ*aceA* of *A. flavus* shows similar virulence to the WT and complemented strains as measured by mortality (*p* = 0.34, Figure 7A). In contrast, deletion of both *crpA* and *crpB* (*ΔcrpAΔcrpB*) resulted in significantly reduced mortality rate compared to the WT and AceA^c^ strains (Figure 7(A)). However, lung fungal burden after 48 h of infection was significantly reduced in both *ΔaceA* (~ 2-fold *p* = 0.0027) and *ΔcrpAΔcrpB* (~ 4-fold, *p* = 0.0001) (Figure 7(B)) suggesting a need for AceA in colonization although not to the degree as the transporters. Histological staining with GMS (stains fungal elements black) or H&E (stains neutrophils dark purple) revealed large fungal lesions with extensive neutrophil infiltration in *A. flavus* WT and *AceA^c^*, smaller fungal lesions with less extensive neutrophil infiltration in Δ*aceA* and very few small fungal lesions with weak neutrophil infiltration in Δ*crpA*Δ*crpB* (Figure 7(C)). These results support a major role for the Cu transporters CrpA and CrpB and a lesser role for AceA in virulence. These results also correlate well with the greater *in vitro* Cu-sensitivity of the Δ*crpA*Δ*crpB* strain compared to Δ*aceA* (Figure 5).

## Discussion

Although a scan of the literature indicates that numerous fungi may crossover to infect both plants and animals, this is a very rare event [37]. However, a prominent fungal exception to this rarity is *A. flavus* which not only causes toxic seed diseases but also is a common agent of keratitis as well as a frequent pathogen of immunosuppressed patients [26,38]. This fungus is common in agricultural soils and hard to eliminate from that environment due to formation of resistant overwintering structures called sclerotia. The fungus is also hard to treat, both as a seed pathogen and human pathogen due to a limited ability to apply fungicides to edible portions of plants and its emerging resistance to several antifungals [39]. Thus, it is important to identify *A. flavus* genes and processes important for virulence in both plant and animal disease.

Several recent studies have shown that fungal nutrient and element acquisition pathways may be critical for pathogenicity of both plant and human pathogens [40,41]. For example, all living organisms utilize Cu as cofactor of enzymes functioning in many cellular biochemical processes [3]. However, hyperaccumulation of Cu generates toxic hydroxyl radicals that pose severe cellular damage [42]. Thus, Cu homeostasis, including Cu uptake, utilization and detoxification is tightly controlled by cells. In *A. fumigatus*, six putative Cu transporters have been identified [15,23], where two are required for Cu import (CtrA2, Afu6g02810 and CtrC, Afu2g03730 [23], one for Cu export (CrpA, Afu43g12740 [15], one for melanin synthesis (CtpA, Afu4g12620 [25] and two others remain characterized (Afu3g13660 and Afu3g08180)[43]. A double deletion of *ctrA2* and *ctrC* showed decreased superoxide dismutase and laccase activity, but did not have an impact on fungal virulence [23]. In contrast, deletion of CrpA resulted in a significant reduction in virulence in murine models of invasive aspergillosis [15]. Recent studies in *A. fumigatus* and *A. nidulans* have demonstrated that the Cu binding transcription factor AceA is responsible for sensing excess Cu, and induces expression of *crpA* as a detoxification mechanism [15,22]. Our study here demonstrates a similar mechanism exists in *A. flavus*, however with the twist of the presence of an additional AceA regulated Crp protein where the two proteins appear to play a redundant function in protecting the fungus from excess Cu. Previous studies have reported on gene duplications of heavy metal transporters in yeast species [44,45]. While in some cases the gene duplications lead to neofunctionalization [44], in others they can lead to extreme halotolerance [45]. As we find an increased Cu tolerance of *A. flavus* and *A. parasiticus* compared to other *Aspergillus* species that only harbor one Crp-type ATPase protein (Figures 1 and 2), we speculate that in *A. flavus* and *A. parasiticus* a gene duplication contributed to increased Cu-export efficiency and not to a neofunctionalization. Nevertheless, this may not be the only factor as two other species, *A. carbonarius* and *A. brasiliensis*, also grew on high concentrations of Cu without a Crp-type ATPase gene duplication. Possibly a higher expression level of *crp* gene or Crp-type ATPase activities might exist in these two fungi.

In *Saccharomyces cerevisiae*, cells are protected from the toxic effects of excess Cu by protein metallothioneins that contain cysteine rich residues, detoxifying Cu by coordination with multiple Cu ions [46]. While metallothioneins appear to play the major role in Cu resistance in *S. cerevisiae*, a putative metallothionein, CrdA, identified in *A. nidulans* did not exhibit a role in Cu detoxification [22]. The homolog in *A. fumigatus*, CmtA, is not expressed in either Cu deficient or Cu excess conditions, nor is it regulated by AceA, thus suggesting it is also not important in Cu homeostasis in that fungus [15]. Instead of metallothioneins, the P-type Cu ATPase, Crp1 in *Candida albicans* and CrpA in *A. fumigatus/nidulans*, was found to be responsible for Cu resistance [15,19,22]. Crp1/CrpA proteins contain GMXCXXC and CXXC consensus motifs that bind free Cu iron or receive chaperone-bound Cu and pump Cu out of the cell through a transmembrane channel [2,19,22]. In this study, we found that most of the analyzed *Aspergillus* species including *A. nidulans* and *A. fumigatus* possess just one CrpA homolog, while one or more redundant copies of the CrpA homolog were identified in *A. flavus, A. oryzae* and *A. parasiticus*, likely leading to the high copper tolerance of these three fungi (Figures 1 and 2, Supplementary Figure S1). Other species (*A. sydowii, A. glaucus, A. arachidicola, A. calidoustus*, and *A. cristatus*) also harbour additional copies of the CrpA homolog (Figure 2) suggesting that they may also exhibit increased Cu tolerance.

As Cu is an essential trace element for most living organisms, and can also be toxic to cells due to its redox properties, Cu is an effective antimicrobial agent and also used by mammalian host cells as defense mechanisms [12]. In the phagolysosome, where macrophages compartmentalize microbial invaders, reactive oxygen intermediates (ROIs), reactive nitrogen intermediates (RNIs) and low pH conditions are generated to kill pathogens. To attack the invading microbes with excess Cu at the infection site of lung, macrophages elevated the Cu level by inducing expression of both ATP7A and Ctr1 [7,12]. A significant induction of Ctr1 was found in *A. fumigatus* challenged macrophages, indicating that Cu is mobilized against the fungal invader [15]. The accumulating Cu works with ROIs, generating the toxic hydroxyl radicals and killing the invading pathogens. Thus, loss of either *aceA* or *crpA* decreased virulence of *A. fumigatus* in a murine model of invasive aspergillosis. Here we found that while Δ*crpA*Δ*crpB* showed decreased murine virulence in all measurable parameters (mortality, fungal load and histology), Δ*aceA* only showed an intermediate attenuated virulence characterized by a reduction in fungal load and slightly less invasive growth as evidenced by histology. This result suggests that some CrpA/CrpB activity remains in the Δ*aceA* mutant. An additional line to pursue in the future could include assessing virulence of these *A. flavus* mutants in a chronic granulomatous disease (CGD) murine model. CGD patients, particularly susceptible to infections by *Aspergillus*, are impaired in ROI generation [47] and possibly at differential risk to strains of *Aspergillus* unable defend from Cu generated ROI.

Although many studies have shown that the Cu detoxification system plays important roles in virulence of human pathogens [15,48,49], to date there hasn’t been a study of a role for CrpA in a plant pathogenic fungus, nor is there any evidence the plant cells use Cu as a defense mechanism. The only study investigating any role of a Cu transporter in a plant pathogen is where a Cu golgi transporter, BcCCC2, in the pathogen *Botrytis cinerea* was found important for virulence in that fungus [24]. Here we found no role for AceA or CrpA/B in virulence of *A. flavus* on host corn seed. Whether this is due to a general lack of a Cu based defense system in plants or a consequence of the somewhat dormant nature of seed responses to pathogens is unknown. However, not surprisingly, we found that the *aceA* and *crpA* double mutants failed to colonize on maize kernel seeds treated with the Cu fungicide.

In conclusion, we identified two copies of the Cu exporter P1-type ATPase in *A. flavus*, leading to a greater tolerance of Cu in this species than other Aspergilli. Both *crpA* and *crpB* are transcriptionally regulated by the Cu-fist TF AceA and both need to be deleted to detect sensitivity to Cu or ROI species. Further, our study illustrates that genes/processes important for virulence of this crossover pathogen in the animal Kingdom may not be a factor in infections of the plant Kingdom.

## Materials and methods

### Strains and culture conditions

All *Aspergillus* strains used in this study are listed in Table 1. *Aspergillus* strains were grown on glucose minimal medium (GMM) [50] with 1 μM Cu at 37°C or 30°C with appropriate supplements as indicated. For *argB* auxotrophs, 1 g/L arginine were added in the growth medium. For *pyrG* auxotrophs, 5 mM uridine and 5 mM uracil were added in the medium. Liquid GMM with 0.5% yeast extract was used to extract genomic DNA. Conidia were harvested freshly in 0.01% Tween 80, and were counted using a hemocytometer. For growth assay, 2 μL 10^6^ conidia/mL of indicated strains were spotted onto the solidified GMM (Noble Agar, Difco, BD) plates under different Cu concentrations. For Cu tolerance assays, non-EDTA trace elements were used.

**Table 1.** *Aspergillus* strains used in this study.

Name of strain	Genotype	Source
NRRL 3357	*A. flavus* Wild-type	Keller lab
GD4.4	*A. flavus* Wild-type	Keller lab
SRRC 28	*A. flavus* Wild-type	Keller lab
TXZ118.2	*A. flavus* Wild-type	Keller lab
PC70	*A. flavus* Wild-type	Keller lab
PC42	*A. flavus* Wild-type	Keller lab
Af293	*A. fumigatus* Wild-type	Keller lab
SRRC44	*A. fumigatus* Wild-type	Keller lab
SRRC46	*A. fumigatus* Wild-type	Keller lab
SRRC43	*A. fumigatus* Wild-type	Keller lab
SRRC 2006	*A. fumigatus* Wild-type	Keller lab
CEA10	*A. fumigatus* Wild-type	Keller lab
FGSC4A	*A. nidulans* Wild-type	Keller lab
CBS 513.65	*A. clavatus* Wild-type	Keller lab
CBS 506.65	*A. zonatus* Wild-type	Keller lab
DTO 11-B6	*A. carbonarius* Wild-type	Keller lab
CBS 101740	*A. brasiliensis* Wild-type	Keller lab
CBS 172.66	*A. aculeatus* Wild-type	Keller lab
CBS 113.46	*A. niger* Wild-type	Keller lab
NIH 2624	*A. terreus* Wild-type	Keller lab
SRRC 2104	*A. oryzae* Wild-type	Keller lab
SRRC 2098	*A. oryzae* Wild-type	Keller lab
SRRC 266	*A. oryzae* Wild-type	Keller lab
SRRC 2103	*A. oryzae* Wild-type	Keller lab
SU-1	*A. parasiticus* Wild-type	Keller lab
ATCC 56774	*A. parasiticus* Wild-type	Keller lab
SRRC 141	*A. parasiticus* Wild-type	Keller lab
SRRC 1039	*A. parasiticus* Wild-type	Keller lab
SRRC 143	*A. parasiticus* Wild-type	Keller lab
SRRC 164	*A. parasiticus* Wild-type	Keller lab
TJW 149.27	*pyrG1*, Δ*ku70::AfpyrG*	[57]
TJES20.1	*pyrG1*, Δ*ku70*, Δ*argB::AfpyrG*	[58]
TXZ 21.3	*pyrG1*, Δ*ku70*, Δ*argB*	[58]
TJES19.1	*pyrG1*, Δ*ku70,*	[58]
TKY6.1	*pyrG1, Δku70, ΔcrpA::argB*	This study
TKY6.3	*pyrG1, Δku70*, Δ*argB::AfpyrG, ΔcrpA::argB*	This study
TKY7.1	*pyrG1, Δku70*, Δ*argB::AfpyrG, ΔcrpB::argB*	This study
TKY11.4	*pyrG1*, Δ*ku70, ΔcrpA::argB, ΔcrpB::AfpyrG*	This study
TKY5.1	*pyrG1*, Δ*ku70*, Δ*aceA::argB*	This study
TKY5.3	*pyrG1*, Δ*ku70*, Δ*argB::AfpyrG*, Δ*aceA::argB*	This study
TKY10.3	*pyrG1*, Δ*ku70, ΔaceA::argB, aceA::AfpyrG*	This study
TKY20.2	*pyrG1*, Δ*ku70, gpdA(p)::crpA::AfpyrG*	This study
TKY21.1	*pyrG1*, Δ*ku70, gpdA(p)::crpB::AfpyrG*	This study
TKY22.4	*pyrG1, Δku70, ΔaceA::argB, gpdA*(*p)::crpA::AfpyrG*	This study
TKY23.3	*pyrG1, Δku70, ΔaceA::argB, gpdA*(*p*):: *crpB:: AfpyrG*	This study

### Strain construction

*Aspergillus flavus* gene deletion and transformation experiments were conducted following previously described protocols [51]. For the disruption of *crpA, crpB* and *aceA*, a homologous recombination strategy was used to replace each gene with *A. flavus argB* in the parental strain TJES20.1 and TXZ21.3 strain protoplasts. The double-joint fusion PCR was performed to generate the disruption constructs [52]. All primers used for gene deletion are listed in Table S2. The primers P1 and P3 were used to amplify the 5ʹ flanking fragment of the gene in questions, while primers P4 and P6 were used to amplify the 3ʹ flanking fragment of the gene. *Aspergillus flavus argB* was amplified from genomic DNA using primers argB/F and argB/R. The nested primers P2 and P5 were used to generate entire disruption constructs. The primers *crpB*/P1 and *crpB-pyrG*/P3 were used to amplify *crpB* 5ʹ flanking fragment, while primers *crpB-pyrG*/P4 and crpB/P6 were used to amplify *crpB* 3ʹ flanking fragment. The nested primers *crpB*/P2 and *crpB*/P5 were used to generate entire *crpB* disruption construct. To generate a Δ*crpA*Δ*crpB* mutant, the purified fusion PCR constructs were co-transformed into TKY6.1 (*pyrG* auxotrophs) strain protoplasts.

*crpA* and *crpB* were each overexpressed, driven by the *A. nidulans gpdA* promoter, at their native loci using double-joint PCR constructs. The marker gene *A. fumigatus pyrG* was used, and was amplified with the primers PyrG/F and PyrG/R from *A. fumigatus Af*293 genomic DNA. The *gpdA* promoter amplified from *A. nidulans* gemonic DNA was inserted directly upstream of the ATG start site. The *crpA* 5ʹ flank was amplified with primers *crpA*/P1 and OE*-crpA*/P3, and the first 1.0 kb fragment of the *crpA* coding region was amplified with primers OE*-crpA*/P4 and OE*-crpA*/P6. The 5ʹ and 3ʹ fragments for the *crpB* overexpression constructs were amplified with the primers *crpB*/P1&OE*-crpB*/P3 and OE*-crpB*/P4&OE*-crpB*/P6, respectively. The nested primers *crp*/P2 and OE*-crp*/P5 were used to generate entire overexpression constructs. The purified fusion PCR constructs were co-transformed into TJES19.1 and TKY5.1 protoplasts.

To generate an *aceA^C^* complemented strain, the *A. fumigatus pyrG* selective marker amplified from its genomic DNA was used. The *aceA^C^* complemented cassette was inserted at the ku70 loci using double-joint PCR constructs. A 3.2-kb PCR product (1.7-kb *aceA* coding sequence, 1.2-kb upstream sequence and 0.3-kb *aceA* terminator region) was amplified from *A. flavus* wild-type genomic DNA using primers *aceA*-CM/F and *aceA*-CM/F, and the two 1-kb flanking fragments of *ku70* was amplified using primers KU70-CM/P1& KU70-CM/P3 and KU70-CM/P4& KU70-CM/P6, respectively. The nested primers KU70-CM/P2 and KU70-CM/P5 were used to generate the entire complemented construct. The purified fusion PCR constructs then were transformed into Δ*aceA* 1-1 to create Δ*aceA* complemented strains TKY10.3. All transformants were confirmed by diagnostic PCR and Southern analysis (Figure S3B). For the Southern analysis, both the 5ʹ and 3ʹ flanking fragment were used as probes.

### Phylogenetic analysis

For phylogenetic analysis, BLASTP [30] hits with an e-value below 1^−20^ against *Aspergillus* species derived from the NCBI database were aligned using MAFFT (Katho et al, 2002). Phylogenetic analysis was performed using fasttree (Price et al., 2009) using the extracted E1-E2 domain from all identified ATPAses. Bootstrap analysis was performed with 1000 replicates.

### Pathogenicity assays on corn

Fungal infection was performed using previously described methods [53,54]. The corn kernels were incubated with 200 μL 10^6^ conidia/mL of indicated strains in a 29°C incubator with a 12-h-light/dark photoperiod for 5 days. Four replicates were conducted for each strain. After incubation, the infected kernels were collected in 2.5 mL methanol to remove the conidia. 100 μL spore suspension was removed for spore amount quantification. Two V of chloroform was added into the vials, and incubated in the dark overnight for aflatoxin extraction. Two mL of the extract was removed, dry down, and resuspended in 1 mL 80:20 water:acetonitrile. HPLC analysis was used for AF analysis according to the published protocol[53].

For application of Cu fungicide, kernels were pretreated with 2.4 g/L Cu powder (wettable powder Champion (Nufarm Americas Inc., IL), and the infection assay was carried out as described above.

### RNA extraction and northern analysis

For Northern expression analysis, all strains at 3 × 10^6^ conidia/L were incubated in 30 mL of liquid GMM without Cu in triplicate at 37°C and 200 rpm shaking for 24 h. To one-half of the cultures, Cu was added to a final concentration of 200 μM for 1 h before harvesting. Mycelia were collected and washed with sterile water, frozen in liquid nitrogen and lyophilized 24 h. Total RNA was extracted with QIAzol Lysis Reagent (QIAGEN), according to the manufacturer’s protocol. Northern analysis was performed as previously described[55]. Probes for Northern analysis were constructed using the primers listed in Table S1 and labeled with dCTP α^32^P.

### Copper quantification

To quantify Cu from the *A. flavus* mycelium, all strains at 5 × 10^6^ conidia/L were incubated in 50 mL of liquid GMM without Cu in four replicates at 37°C and 200 rpm shaking for 12h, then Cu was added to a final concentration of 50 μM for 12 h before harvesting in one half of the treatments. The quantification of copper from the indicated strains was carried out according to our previously publication [15], with the incorporation of sulfur as a mass index, as previously reported, in order to increase accuracy [56].

### Animal studies

Six-week-old female ICR mice were immunocompromised by subcutaneous injection with cortisone acetate (300 mg/kg) 3 days prior to infection, on the day of infection, and 3, 7, and 11 days post-infection. The mice were infected intranasally with 5 × 10^5^ dormant spores, which had been suspended in 20 μL of PBS plus 0.2% Tween 20 (10 μL in each nostril). Survival was monitored for up to 21 days. For histopathology, mice were sacrificed two days after infection and their lungs were removed for histological staining with Grocott’s methenamine silver stain (GMS; fungal staining, counter stained with Light Green SM solution) and hematoxylin and eosin (H&E; tissue and nuclear staining). For fungal burden, infected mice were sacrificed on the second day post infection, their lungs were removed and homogenized, and the homogenates were plated on YAG. The plates were incubated at 37°C for 24 h, and the numbers of colony forming unit (CFU) were counted. Experiments were ethically approved by the Ministry of Health (MOH) Animal Welfare Committee, Israel.

### Statistical analysis

All data were presented as the means **± **standard deviation (SD). Statistical and significance analysis were performed using the GraphPad Prism 5. The statistical differences for mouse survival were calculated using the Log-Rank (Mantel-Cox) test and for organ fungal load using the unpaired t test. One-way ANOVA and the least significant difference (LSD) tests was used to determine significant differences among group means. A *p*-value less than 0.05 was recognized as statistically significant.
