# Supplementary material for: Contribution of ATPase copper transporters in animal but not plant virulence of the crossover pathogen Aspergillus flavus
Source: Virulence. 2018 Aug 23;9(1):1273–86. doi: 10.1080/21505594.2018.1496774 (PMC6177249; doi:10.1080/21505594.2018.1496774)
Supplement: Supplemental Material [file kvir-09-01-1496774-s001.docx]

**Supplemental Figures**

**
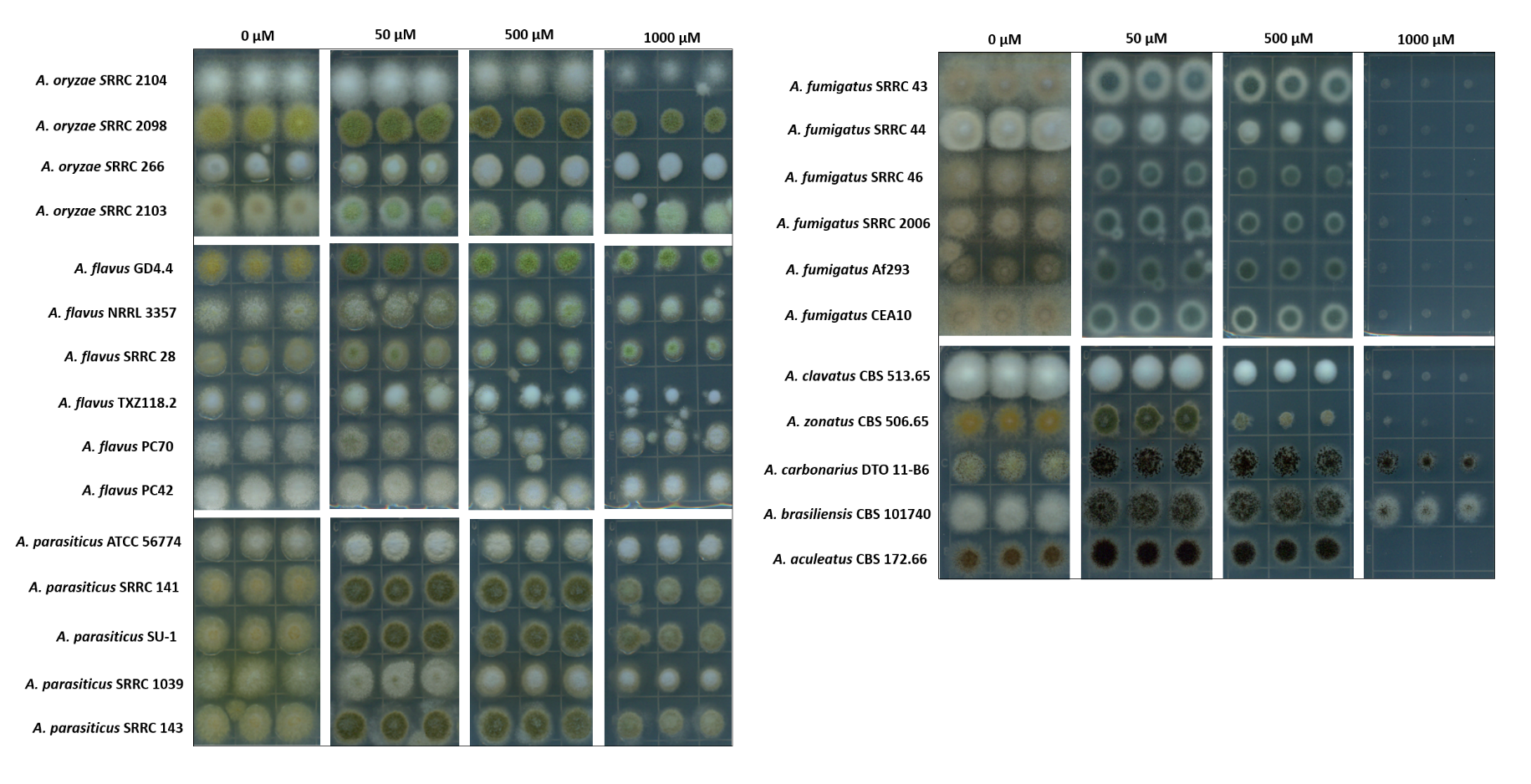
**

**Figure S1: Growth phenotype of additional *Aspergillus* strains on different Cu concentrations.** 2000 spores of indicated *Aspergillus* strains grown on solidified GMM under indicated Cu concentrations for 72 h at 30 °C.

**
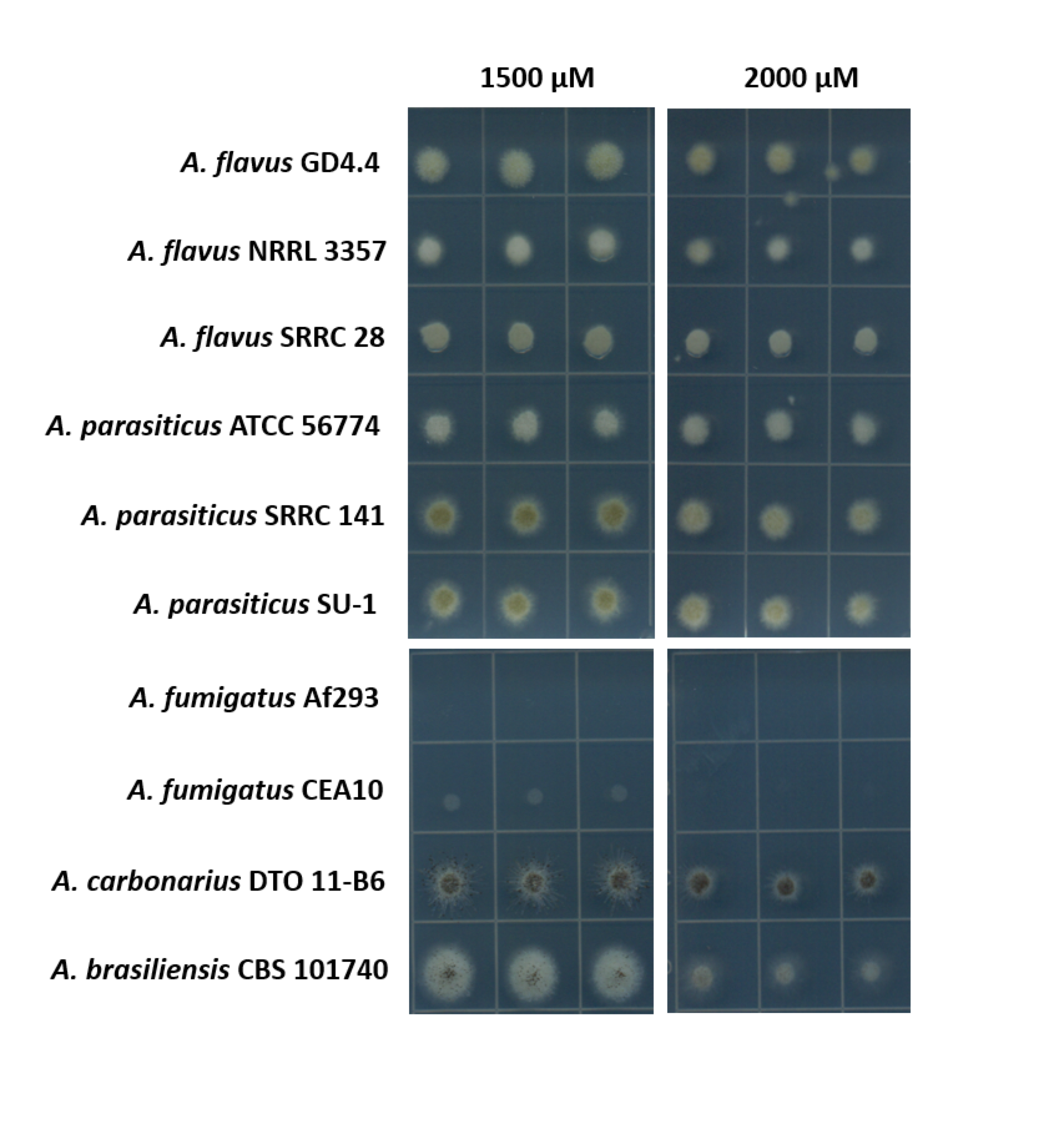
**

**Figure S2: Growth phenotype of additional *Aspergillus* strains on different higher Cu concentrations.** 2000 spores of indicated *Aspergillus* strains grown on solidified GMM under indicated Cu concentrations for 72 h at 30 °C.

**
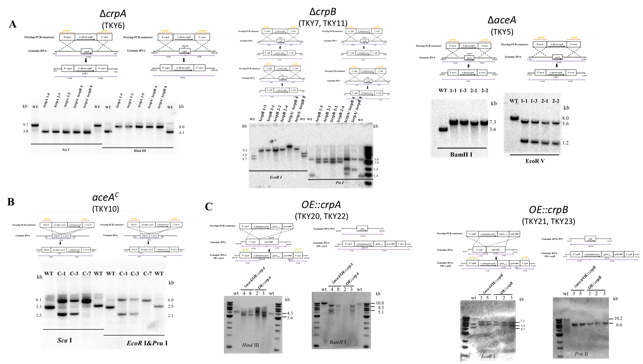
**

**Figure S3: Deletion strategy and Southern analysis of the mutants used in this study.**

(A) Deletion strategy and southern blot analyses for ∆*crpA,* ∆*crpB* and ∆*aceA* strains.

(B) Complement strategy and southern blot analyses for *aceA^C^* complemented strains

(C) Deletion strategy and southern blot analyses for OE::*crpA*, ∆*aceA*/OE::*crpA,* OE::*crpB* and ∆*aceA*/OE::*crpB.*


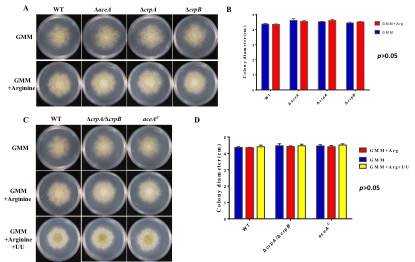


**Figure S4: Impact of arginine, uridine and uracil on the growth of mutants that do not exhibit a marker gene effect.**

(A) Independent Δ*aceA*, Δ*crpA* and Δ*crpB* transformants were compared to the wild type (WT) on GMM media with and without supplementation of arginine. After 4 days, radial growth was measured (B).

(C) The Δ*crpA*/Δ*crpB* and *aceA^C^* strains were also compared on GMM media with and without supplementation of arginine, uridine and uracil (UU). After 4 days, radial growth was measured (D).

*p*>0.05 indicates not significant differences.


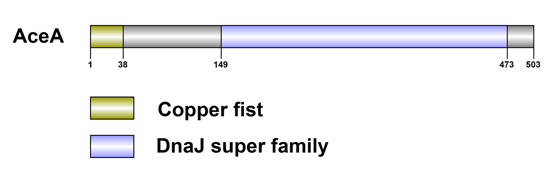


**Figure S5:** Domains analysis of AceA. Domains of AceA was characterized by NCBI Identify Conserved Domains, and software DOG 2.0 were used to visualize protein domains.


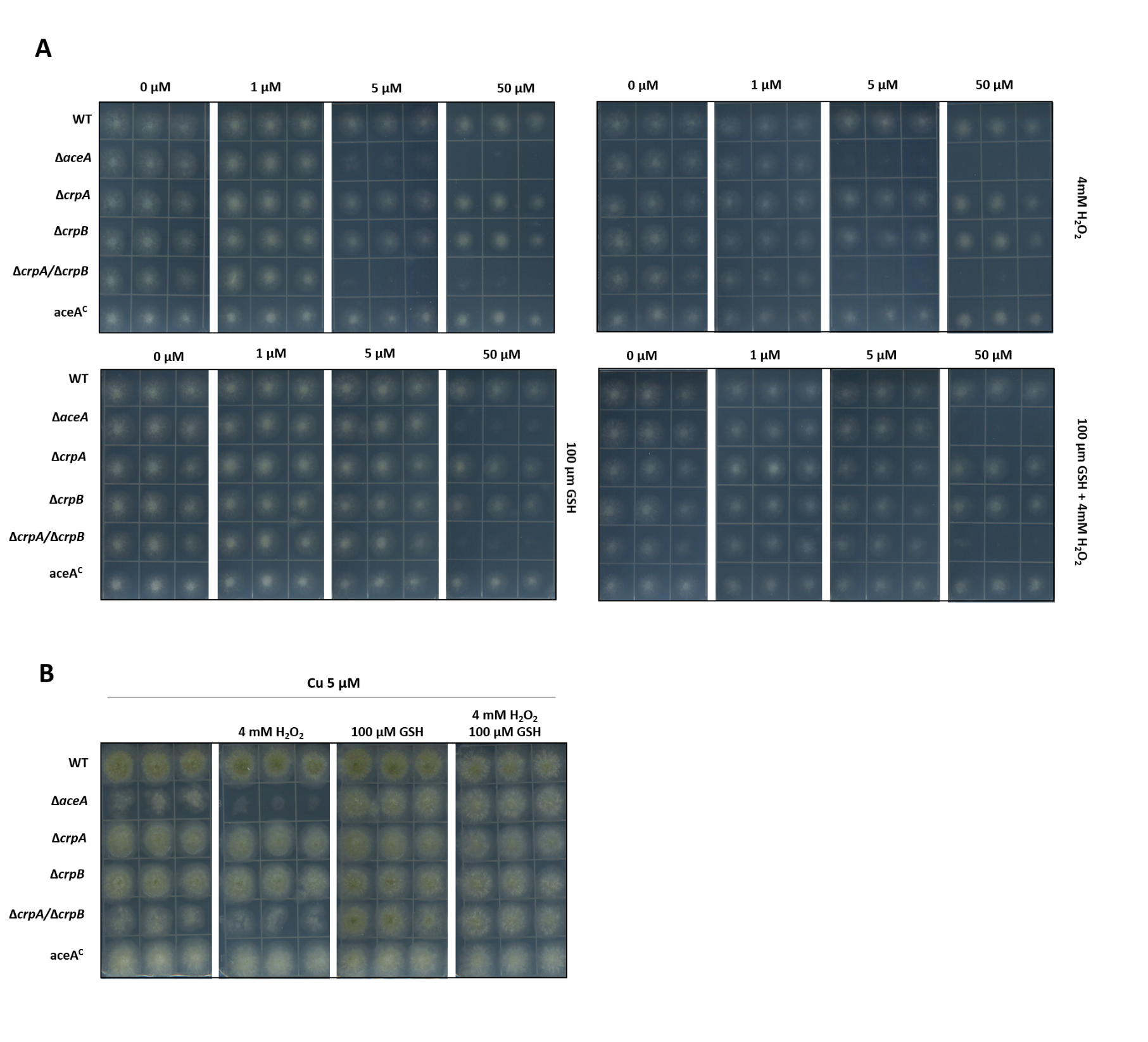


**Figure S6: ROS stress induced by hydrogen peroxide increases Cu toxicity in Δ*aceA* and double Δ*crpA/crpB* mutants.** A. Growth phenotypes of indicated strains on solidified GMM under indicated concentration of Cu plus supplements as indicated for 48 h at 30 °C. B. Growth phenotypes of the same strains under a concentration of Cu of 5 µM plus supplements as indicated for 72 h at 30 °C. H_2_O_2_ = Hydrogen peroxide, GSH = L-glutathione.

**Supplemental tables**

**Table S1 Primers used in this study.**

| Primers | Sequence (5’-3’) | Application |
| --- | --- | --- |
| *crpA*/P1 | TTTACTCGGCGACAAGGCAG | *crpA* deletion and probe |
| *crpA*/P3 | CCCCTTCTACCGAACTCATCACCACCGGGATATGGGCAGTTTAGAAGTATAG |  |
| *crpA*/P4 | CCGTTGGTGCCCGCATTCACATGTCACGGGTGAATGGTTGTTTATATGGG |  |
| *crpA*/P6 | CCACGGTAACACCCATCACC |  |
| *crpA*/P2 | AGACACGCGACAAGCGATGC |  |
| *crpA*/P5 | ACCCGAGAAGTCCGAACCTC |  |
| *crpA*-ORF/F | GTAAAGTCGGCTCAGGGTC | *crpA* mutant screen/Northern probe |
| *crpA*-ORF/R | CAACCATAACTGTCTCACCC |  |
| *crpB*/P1 | TCGGCTATTTGTAAGATGTCG | *crpB* deletion and probe |
| *crpB*/P3 | TTCTACCGAACTCATCACCACCGGGACAGGGCAGGTTCTAGTTGG |  |
| *crpB*/P4 | TGGTGCCCGCATTCACATGTCACGGGATGTTATGGGTCAATTCTGCC |  |
| *crpB*/P6 | AGATCGTCACCAAAGTCCCT |  |
| *crpB*/P2 | TCAGGTCCCGAGCACTAAC |  |
| *crpB*/P5 | CCGAAACGGATACAACCTG |  |
| *crpB*-ORF/F | TTTGTGCCGATTATTACGCTG | *crpB* mutant screen/Northern probe |
| *crpB*-ORF/R | CCTGTGGGAAGAAAGAACCT |  |
| argB/F | TCCCGGTGGTGATGAGTTC | *crp* deletion |
| argB /R | CCCGTGACATGTGAATGCG |  |
| PyrG/F | GCCTCAAACAATGCTCTTCACCC | *crpA&crpB* deletion construct |
| PyrG/R | GTCTGAGAGGAGGCACTGATGC |  |
| *crpB*-pyrG-P3 | GGGTGAAGAGCATTGTTTGAGGCCAGGGCAGGTTCTAGTTGG |  |
| *crpB*-pyrG-P4 | GCATCAGTGCCTCCTCTCAGACATGTTATGGGTCAATTCTGCC |  |
| *aceA*/P1 | GGGCTGACTTCCGGGATTAC | *aceA* deletion and probe |
| *aceA*/P3 | CCCCTTCTACCGAACTCATCACCACCGGGATATCGCGGTTACTGGGTAGC |  |
| *aceA*/P4 | CCGTTGGTGCCCGCATTCACATGTCACGGGGCGCAGGTTTTCTGGCTTTG |  |
| *aceA*/P6 | ACCTCTCCCACACCGATTGG |  |
| *aceA*/P2 | TGTGGAGCAGACCACGAAGC |  |
| *aceA*/P5 | GGTACCGGTACCTACCTTCC |  |
| *aceA*-ORF/F | TACCTTCCCGACCACGACC | *aceA* mutant screen |
| *aceA*-ORF/R | CGAAAGGCTCGTCGTAGGG |  |
| *aceA*-CM/F | ACTTTTACGGTGACAAACCTCCCACTATAGCTTCAGCACGAGGGAC | *aceA* complementation construct |
| *aceA*-CM/R | AGAGGGTGAAGAGCATTGTTTGAGGCGAGAGTATTAGGCAGTAGTGG |  |
| KU70-CM/P1 | TTGACTCGGTTACTAATGACG | *aceA* complementation construct |
| KU70-CM/P3 | TAGTGGGAGGTTTGTCACCG |  |
| KU70-CM/P4 | CACGCATCAGTGCCTCCTCTCAGACTCCTAGGTGTCTGCCTGTCG |  |
| KU70-CM/P6 | GCTCATGGGCAGGCTGTGG |  |
| KU70-CM/P2 | AACTCAATACCGCCCTCAAC |  |
| KU70-CM/P5 | ACCGATCAATGACACTCCAC |  |
| OE-*crpA*/P3 | GGGTGAAGAGCATTGTTTGAGGCTATGGGCAGTTTAGAAGTATAG | *crpA* overexpression construct |
| OE-*crpA*/P4 | AGCTACCCCGCTTGAGCAGACATCACATGTCGCCTATATCAGATCAC |  |
| OE-*crpA*/P5 | GTCACGCCGATTTGTTCTAC |  |
| OE-*crpA*/P6 | TATGCTCGGAGGGTGGTAG |  |
| OE-*crpB*/P4 | AGCTACCCCGCTTGAGCAGACATCACATGGTCACAGGTAATCAGCC | *crpB* overexpression construct |
| OE-*crpB*/P5 | ACTTTGCCCACGCAGGAGC |  |
| OE-*crpB*/P6 | GAGACCCTTGATGATTCCTG |  |
| GpdA/F | TGCGGAGAGACGGACGGAC | *gpdA* promoter |
| GpdA/R | GTGATGTCTGCTCAAGCGG |  |
